# Lymphoma in adolescents and young adults: Navigating a path forward together

**DOI:** 10.1002/jha2.793

**Published:** 2023-09-29

**Authors:** Andrew M. Evens, Kara M. Kelly

**Affiliations:** ^1^ Rutgers Cancer Institute of New Jersey Robert Wood Johnson Medical School and Rutgers Health New Brunswick New Jersey USA; ^2^ Roswell Park Comprehensive Cancer Center University at Buffalo Jacobs School of Medicine and Biomedical Sciences Buffalo New York USA

Lymphoma that occurs in adolescents and young adults (AYA) between ages 15 and 39 years is associated with unique incidence, race, and gender distribution [1, 2]. Furthermore, improvement in survival has lagged behind advances seen in children and older adults with lymphoma [3]. Contributing factors are complex, including disease biology, social factors, and access to appropriate clinical care. Historically, the management of common lymphomas in this age group, including Hodgkin lymphoma and mature B‐cell lymphomas, has diverged considerably within pediatric and adult oncology paradigms [4]. In addition, there are only two existing consensus AYA survivorship guidelines, which are the Children's Oncology Group Long‐Term Follow‐up Guidelines (COG LTFU) and National Comprehensive Cancer Network (NCCN AYA) guidelines [5, 6]. There is disagreement between the guidelines on the link between treatment exposures and late effects, which patient populations should be screened, the screening tests to be used, and the testing time interval. Concerted cross‐disciplinary collaboration is required to understand the nature of these disparities better, increase harmonization of guidelines, and identify unified strategies to improve outcomes for AYAs.

Recognizing this unmet need, the Lymphoma Research Foundation (LRF) began an initiative to engage and support the AYA research community, patients, and their families. In 2015, LRF convened an AYA Symposium with clinicians and basic scientists from pediatric and adult disciplines to examine the state of the science for AYA lymphoma, precisely review the gaps in research for this population, and discuss the unique challenges and treatment/disease burdens for the AYA lymphoma population [7]. Before this event, no formal cross‐disciplinary scientific collaborations had occurred.

A summary of the symposium proceedings was published in Blood Advances in October 2017 [4]. This publication compared the pediatric and adult approaches to lymphoma management for Hodgkin lymphoma, mature B‐cell lymphomas, and anaplastic large cell lymphoma and found that lymphoma treatment for AYA is not dictated by empiric evidence specific to age group but rather by community referral patterns, individual physician preference, and treatment location. Furthermore, the publication highlighted a knowledge gap surrounding AYA lymphoma biology, care delivery, and therapeutic efficacy. As a result of low rates of clinical trial enrollment, systematically generated evidence is lacking. Additionally, AYA patients, who are often transitioning between pediatric and adult care, are especially vulnerable to lapses in survivorship care.

With the findings of the original Symposium in hand and preliminary results from a range of research funded by the LRF, the first AYA Consortium meeting was held in 2019. The meeting involved panel discussions describing the latest findings in AYA lymphoma, the development of blueprints for subsequent AYA lymphoma research, and drafting an action plan for the Consortium. Blueprints included strategies for understanding features of AYA biology, immunobiology, and epidemiology, guiding clinical trials and drug development, characterizing ideal care delivery and patient outcomes, and better understanding survivorship and late effects. The action plan outlined the need for research to define AYA lymphoma and to support a focus on AYA lymphoma as a distinct patient group.

Subsequently, in June 2022, pediatric and adult oncology physicians and scientists from more than 40 North American academic and medical institutions, federal agencies, pharmaceutical companies, and patient advocates were brought together in Jersey City, New Jersey, for the second LRF AYA Lymphoma Workshop. The workshop focused on research and clinical developments in the areas highlighted by the 2019 AYA blueprint, focusing on the needs of AYA patients, including current treatment paradigms and new collaborative clinical trial efforts, epidemiology, care delivery, survivorship, and cutting‐edge scientific research to better understand and serve these patients. Each session of the meeting included both lectures by select expert faculty and interactive panel discussions where audience members could ask questions, learn from colleagues in different disciplines, and work to develop cooperative strategies to improve AYA care.

In this special issue of the *eJHaem* entitled “Lymphoma in adolescents and young adults: navigating a path forward together,” a multitude of experts from the 2022 AYA Lymphoma Workshop review the current status, discuss significant new developments in the field, and provide recommendations for scientific prioritization, including: (1) the pathobiology of select rare AYA lymphomas; (2) scientific techniques in AYA lymphomas; (3) current directions in the treatment of Hodgkin lymphoma; (4) treatment of mature B‐cell lymphomas in AYAs; (5) overcoming barriers to drug development and enrollment in clinical trials for AYAs with lymphoma; (6) outcomes of AYAs with lymphoma: treatment effects; and (7) disparities and vulnerable AYA lymphoma populations (Table 1).

**TABLE 1:** AYA lymphoma topic reviews.

Author	Title
Steidl C, et al.	The pathobiology of select rare AYA lymphomas
Aoki T, et al.	Scientific techniques in AYA lymphomas
Keller F, et al.	Current directions in the treatment of Hodgkin lymphoma
El‐Mallawany NK, et al.	Treatment of mature B‐cell lymphomas in AYAs
Toner T, et al.	Overcoming barriers to drug development and enrollment in clinical trials for AYAs with lymphoma
Pophali P, et al.	Outcomes of AYAs with lymphoma: treatment effects
Rosenthal A, et al.	Disparities and vulnerable AYA lymphoma populations

Abbreviation: AYA, adolescent and young adults.

The reviews include epidemiologic perspectives on AYA lymphomas and ways that molecular and clinical data can be leveraged to develop a precision approach in less common AYA lymphomas such as nodular lymphocyte‐predominant Hodgkin lymphoma. There is also an assessment of the biology of follicular lymphoma in pediatric and AYA populations and an examination of the biology of primary mediastinal B‐cell lymphomas and AYA posttransplantation lymphoproliferative disorders. Scientific techniques reviewed include whole genome sequencing in common AYA lymphomas and research on the therapeutic importance of tumor microenvironment and single cell biology in lymphoma. Additionally, the importance of circulating tumor DNA for genetic profiling and monitoring of pediatric Hodgkin lymphoma is examined.

There is a joint appraisal of the pediatric and adult experience within Hodgkin lymphoma for early‐stage disease, advanced‐stage, and relapsed/refractory disease for AYAs. The authors highlight the recently presented study, S1826, “Immunotherapy plus combination chemotherapy in treating patients with newly diagnosed stage III‐IV classic Hodgkin lymphoma,” that was conducted via the National Clinical Trials Network (NCTN) as well as a similarly constructed large study in early‐stage Hodgkin lymphoma, AHOD 2131 [8]. These pivotal phase 3 randomized studies include broad and robust collaboration amongst the Children's Oncology Group and all adult oncology cooperative groups [9].

Current progress and unmet needs in mediastinal large‐cell lymphomas are reviewed, and the pediatric mature B‐cell lymphoma landscape and factors impacting outcomes for patients are examined; a more focused discussion regarding the optimal incorporation of novel agents in the management of diffuse large B‐cell lymphoma for AYAs is presented. A collaborative review amongst the Cancer Therapy Evaluation Program (CTEP), biotech, and academia includes an overview of the structure of the NCTN and the identification of methods to overcome barriers to drug approval and the time and resources needed for running clinical trials that include a substantial number of AYA patients.

A review of the AYA outcomes includes the postacute and long‐term complications in AYA lymphomas, the use of multisource data to characterize outcomes after initial Hodgkin lymphoma treatment, the discussion of treatment toxicity and tolerability in AYA, and an examination of survivorship guidelines for AYA lymphomas. Finally, a synopsis of current data on access and disparities in AYA lymphomas, focusing on opportunities for engagement, including specialized AYA models of care, financial toxicity, and disparities in AYA lymphoma, is provided. This review provides an enhanced understanding of the interplay of social and structural determinants of health with features of healthcare, economic, educational, environmental, and community structures.

Altogether, the reviews and associated recommendations in this special issue of *eJHaem* represent critical stepping stones to help navigate a path forward together. With the positive momentum and robust collaborative efforts via the initial AYA Lymphoma Workshops, the LRF recently founded an AYA Lymphoma Consortium [7]. This consortium is the first collaboration of its kind of a broad multidisciplinary effort aimed at advancing the study of AYA lymphomas and improving treatments and care for this patient population, from the point of diagnosis through long‐term survival. There will be coordinated collaboration among laboratory and translational scientists, clinical experts (pediatric and adult), epidemiology, health services, social scientists, and patient advocates. This endeavor will include educational, research, and outreach efforts on lymphoma subtypes that most significantly impact the AYA patient population and will emphasize several essential study areas (Table 2). Collectively, the Consortium will provide a dynamic forum for worldwide collaboration and intellectual exchange on areas of relevance to AYA lymphomas, and the group will identify gaps in knowledge to be prioritized for research funding, optimization of clinical outcomes, and discoveries for AYA lymphomas.

**TABLE 2:** AYA lymphoma consortium of the LRF.

**Key areas of study**
Disease biology, etiology, and diagnosis
Treatment approaches
Drug development and enrollment in clinical trials
Outcomes
Survivorship
Diversity, equity, and inclusion

Abbreviations: AYA, adolescent and young adults; LRF, Lymphoma Research Foundation.

## AUTHOR CONTRIBUTIONS

AE collected and analyzed the data, interpreted the data, and co‐wrote the manuscript. KK collected and analyzed the data, interpreted the data, and co‐wrote the manuscript.

## CONFLICT OF INTEREST STATEMENT

AME: Advisory Board with honorarium (research related): Seattle Genetics, Hutchmed, Incyte, Daiichi Sankyo, Novartis, Abbvie, Pharmacyclics and Epizyme. KMK: Scientific Steering Committee: Merck, and Advisory Board without honorarium: Seattle Genetics. No financial support was necessary for the preparation of this manuscript or acquiring data.

## ETHICS STATEMENT

The authors have confirmed ethical approval statement is not needed for this submission.
